# Albumin for patients with acute large-vessel occlusive stroke undergoing endovascular therapy (ARISE): the protocol of a randomized double-blind trial

**DOI:** 10.3389/fneur.2025.1570184

**Published:** 2025-07-18

**Authors:** Yuanyuan Liu, Xiao Dong, Xuehong Chu, Zhengfei Ma, Tingyu Yi, Changming Wen, Yifeng Liu, Jun Sun, Jing Xu, Wenbo Li, Lei Yang, Benxiao Wang, Lei Shi, Jianqiao Li, Xiaoman Zhang, Chaoqun Li, Wenhuo Chen, Chuanhui Li, Di Wu, Chengbei Hou, Chen Zhou, Ming Li, Yi Xu, Chuanjie Wu, Xunming Ji

**Affiliations:** ^1^Department of Neurology, Xuanwu Hospital of Capital Medical University, Beijing, China; ^2^Department of Neurology, Suzhou Municipal Hospital, Suzhou, Anhui, China; ^3^Department of Neuro-intervention, Zhangzhou Municipal Hospital, Zhangzhou, Fujian, China; ^4^Department of Neuro-intervention, Zhangzhou Affiliated Hospital of Fujian Medical University, Zhangzhou, Fujian, China; ^5^Department of Neurology, Nanyang Central Hospital, Nanyang, Henan, China; ^6^Department of Neurology, Maanshan People's Hospital, Maanshan, Anhui, China; ^7^Department of Neurology, Luoyang Central Hospital Affiliated To Zhengzhou University, Luoyang, Henan, China; ^8^Department of Neurology, Liaocheng Third People's Hospital, Liaocheng, Shandong, China; ^9^Department of Neurology, WanBei Coal-Electricity Group General Hospital, Suzhou, Anhui, China; ^10^Department of Neurology, Si County People's Hospital, Anhui, China; ^11^Department of Neurology, Sui Xi County Hospital, Huaibei, Anhui, China; ^12^Department of Neurology, The First People’s Hospital of Zhengzhou, Zhengzhou, Henan, China; ^13^Department of Neurology, Xihua County People's Hospital, Zhoukou, Henan, China; ^14^Department of Cerebrovascular Disease, Fujian Medical University Union Hospital, Fujian, China; ^15^China-America Institute of Neuroscience and Beijing Institute of Geriatrics, Xuanwu Hospital, Capital Medical University, Beijing, China; ^16^Center for Evidence Based Medicine, Xuanwu Hospital, Capital Medical University, Beijing, China; ^17^Beijing Institute for Brain Disorders, Capital Medical University, Beijing, China

**Keywords:** albumin, neuroprotection, endovascular therapy, acute ischemic stroke

## Abstract

**Background:**

Albumin is a multifunctional plasma protein that is mainly synthesized in the liver and may play a neuroprotective role in treating acute ischemic stroke (AIS). The efficacy of albumin in patients with AIS receiving reperfusion therapy remains unknown.

**Methods:**

ARISE is a multicenter, randomized, double-blind, placebo-controlled, phase 2 study. We will recruit 134 patients aged 18–80 years with AIS due to large-vessel occlusion in the anterior circulation, within 24 h of symptom onset, with an Alberta Stroke Program Early CT Score of 3–10 points and an infarct core volume of ≤100 mL at baseline. Eligible patients will be randomly assigned, on a 1:1 ratio, to undergo endovascular therapy (EVT) and receive albumin therapy (0.5 g/kg; intravenous injection) once daily for 4 days or to undergo EVT and receive placebo therapy once daily for 4 days. The primary efficacy outcome is the change in infarct volume from baseline to day 5.

**Conclusion:**

The ARISE trial will provide valuable evidence on the efficacy and safety of albumin in patients with AIS receiving EVT.

**Clinical trial registration:**

www.clinicaltrials.gov, NCT06538844.

## Background

Stroke is the most common cause of death and disability worldwide, with acute ischemic stroke (AIS) accounting for 80% of all cases (1, 2). Effective reperfusion therapies, such as intravenous thrombolysis and endovascular therapy (EVT), have been widely used in the treatment of AIS patients (3–5). While recanalization of occluded arteries is central to the treatment of AIS, patient prognosis may be further improved with additional treatment options that preserve and enhance brain function through neuroprotection (6, 7). Neuroprotective drugs can freeze the ischemic penumbra and target the ischemic cascade following cerebral infarction, including mechanisms such as oxidative stress and inflammation. This may reduce the inflammatory response, limit hemorrhagic transformation, and improve neurological prognosis after cerebral infarction (8, 9). However, neuroprotection in AIS has a long history of clinical trial failures over the past several decades (9, 10). One possible reason is that many of these drugs were not combined with reperfusion therapy (11, 12). Therefore, it is recommended that previously unsuccessful neuroprotective drugs, especially those supported by strong preclinical research evidence and confirmed to be safe in clinical studies, be reconsidered in the context of reperfusion therapy (9).

Albumin, the predominant protein in plasma, is mainly synthesized in the liver (13). Although the exact molecular mechanisms underlying albumin’s effects remain unclear, preclinical studies have shown its neuroprotective effects in several animal models of cerebral infarction. These effects include reducing cerebral edema through dehydration and increasing cerebral blood flow in ischemic areas (14–16). However, the Albumin in Acute Ischemic Stroke (ALIAS) trial, conducted from 2009 to 2012, did not confirm that high-dose albumin improves neurological prognosis in AIS patients (17). In this study, only 21% of participants received EVT. EVT devices and therapeutic techniques, along with the expansion of indications for EVT and the reduction of barriers to EVT selection through imaging, were not fully developed at the time of the study (17). The fact that neuroprotective treatment with albumin was not combined with efficient reperfusion therapy may have contributed to the failure of the study. Therefore, it is important to reevaluate the neuroprotective role of albumin in the context of reperfusion therapy for AIS.

This protocol describes the rationale and design of the ARISE (Albumin for Patients with Acute Large-Vessel Occlusive Stroke Undergoing Endovascular Therapy) trial, which aims to investigate albumin’s safety and efficacy for AIS patients undergoing EVT.

## Methods

### Ethical approval and informed consent

The clinical trial complies with the ethical guidelines outlined in the Declaration of Helsinki, which governs medical research involving human subjects. The ethics committees and institutional review boards of all participating clinical sites approved the study protocol. Prior to participation, written informed consent will be obtained from all participants or their legal guardians.

### Study design

ARISE is a multicenter, randomized, double-blind, placebo-controlled trial with two parallel (1:1) groups (albumin or placebo), designed to evaluate the safety and efficacy of albumin for patients with AIS receiving EVT. All participants will be followed for 3 months. The study has been registered in www.clinicaltrials.gov (registration number NCT06538844).

### Patient population

Patients will be eligible for enrollment if they have an acute ischemic stroke due to a large-vessel occlusion in the intracranial segment of the internal carotid artery or the M1 or M2 segment of the middle cerebral artery. Eligible patients must be aged 18–80 years, have a modified Rankin Scale (mRS) score of ≤1 the onset of the disease, have a National Institute of Health Stroke Scale (NIHSS) score of ≥6, have an Alberta Stroke Program Early CT Score (ASPECTS) of ≥3, and have ischemic core volume of ≤100 mL. Patients must be enrolled up to 24 h after the onset of stroke symptoms. A complete list of inclusion and exclusion criteria is provided in Table 1.

**Table 1:** Inclusion and exclusion criteria.

Inclusion and exclusion criteria	
Inclusion criteria	Age between 18 and 80 years; Anterior circulation acute ischemic stroke, with acute occlusion of the responsible vessel located in the intracranial segment of the internal carotid artery, or the M1 or M2 segment of the middle cerebral artery; National Institute of Health Stroke Scale (NIHSS) score ≥6; Modified Rankin Scale (mRS) score ≤1 before onset of the disease; Alberta Stroke Program Early CT Score (ASPECTS) ≥ 3 points; Ischemic-core volume ≤100 mL; Patient is treatable within 24 h of symptom onset. Symptom onset is defined as the point in time the patient was last seen well (at baseline) if patients are unable to provide a reliable history, or the point in time when symptoms have started if patients can provide a reliable history. Treatment start is defined as groin puncture; Written informed consent provided by the patients or their legal relatives.
Exclusion criteria	Evidence of intracranial hemorrhage (intracerebral hematoma (ICH), subarachnoid hemorrhage (SAH), epidural hemorrhage, acute or chronic subdural hematoma (SDH)) on the baseline CT or MRI scan; History of congenital or acquired bleeding disorders, coagulation factor deficiency diseases, thrombocytopenic diseases, etc.: Anticipated difficulty in completing endovascular treatment due to vascular tortuosity; Known severe allergy (more severe than skin rash) to contrast agents uncontrolled by medications; Pregnancy and breastfeeding; An episode or exacerbation of congestive heart failure from any cause in the past 6 months; History of heart valve disease complicated by congestive heart failure within the past 6 months; Cardiac surgery with thoracotomy (e.g., coronary artery bypass grafting or valve replacement surgery) within the past 6 months; Acute myocardial infarction in the past 6 months; Signs or symptoms of acute myocardial infarction upon admission, including electrocardiographic findings; Elevated serum troponin concentration upon admission (>0.1 μg/L); Acute arrhythmia (including any tachycardia or bradycardia) with hemodynamic instability (systolic blood pressure <100 mm Hg) upon admission; Acute or chronic lung diseases requiring long-term or intermittent oxygen therapy; Findings on physical examination of any of the following abnormalities: (1) jugular venous distension (jugular venous pulsation >4 cm above the sternal angle); (2) resting tachycardia due to congestive heart failure (heart rate > 100 per/min); (3) third heart sound; (4) abnormal hepatic jugular venous reflux; (5) pitting edema of the lower extremities attributable to congestive heart failure or without apparent cause; (6) rales in both lungs; and (7) or evidence of pulmonary edema, pleural effusion, or pulmonary vascular redistribution on chest X-ray; Known recent or current serum creatinine exceeding 1.5 times the upper limit of normal, or an estimated glomerular filtration rate (eGFR) < 60 mL/min; Refractory hypertension that is difficult to control with medication (defined as systolic blood pressure > 220 mmHg, or diastolic blood pressure > 110 mmHg); Severe chronic anemia (hemoglobin < 75 g/L); History of albumin allergy or known allergy to albumin; Patients with severe mental disorders or dementia who are unable to cooperate in completing informed consent and follow-up content; The expected survival time is less than 90 days (such as comorbidity with malignant tumor or severe systemic diseases); Patients who participated in other interventional clinical studies within 30 days prior to randomization or are currently participating in other interventional clinical studies.

### Randomization

Eligible patients will be randomly assigned (1:1) to receive albumin or placebo via a web-based interactive response system. A minimization process will be used to balance the stratification factors of age (≤ 60 or > 60 years) and baseline ASPECTS (3 to 6 or ≥6) at each medical site.

### Treatments

**Figure 1:**
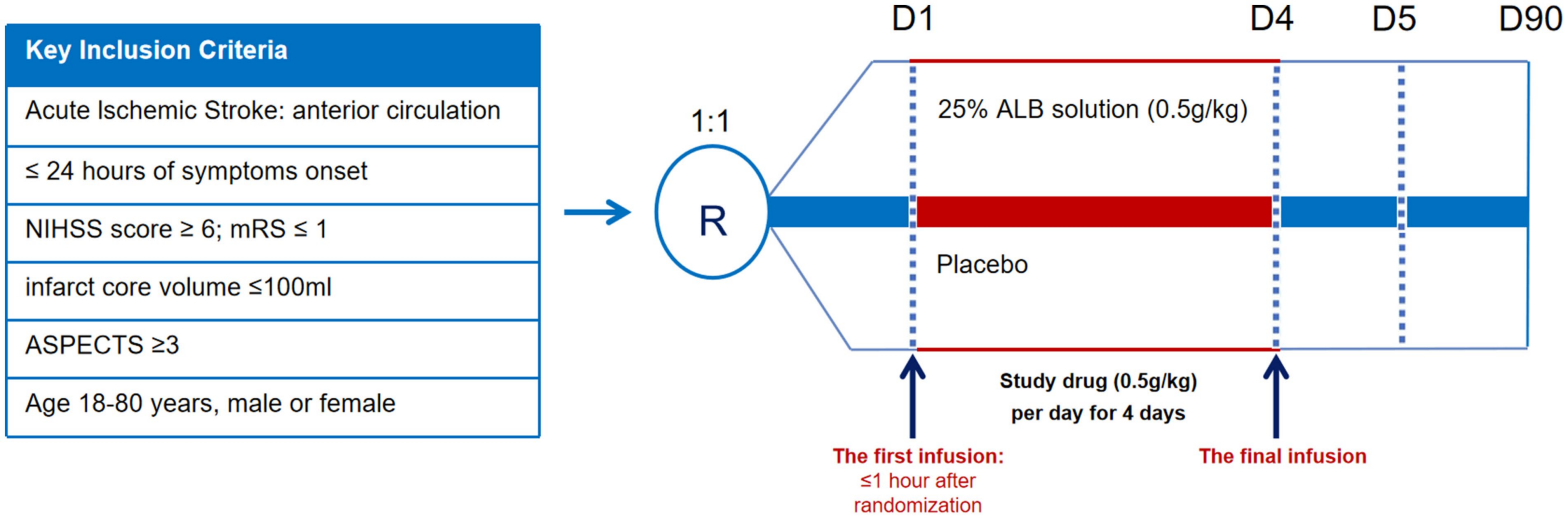
Graphical study design. NIHSS, National Institute of Health Stroke Scale; mRS, Modified Rankin Scale; ASPECTS, Alberta Stroke Program Early CT Score.

After qualifying imaging, patients will undergo EVT using currently available devices as quickly as possible and will receive intravenous thrombolysis if indicated (before or during EVT, at a primary hospital prior to transfer, or at the comprehensive stroke center). The experimental group will receive a 25% albumin solution (0.5 g/kg; maximum dose 150 mL) administered via intravenous infusion once daily for 4 days. The control group will receive an equivalent volume of placebo injection intravenously once daily for the first 4 days (Figure 1). The trial drugs will be manufactured and provided by Shanghai RAAS Blood Products Co., Ltd., Shanghai, China. All researchers and patients will be blinded to treatment allocation. The slightly differently colored study drugs will be concealed. The first infusion will be administered within 1 h after randomization. The steering committee will make recommendations for concurrent treatments. Equal care following the American Heart Association/American Stroke Association guidelines for AIS during admission will be provided for all patients.

### Study outcomes

The primary efficacy endpoint is the change in infarct volume from baseline (as measured by computed tomography perfusion (CTP)) to day 5 (as measured by fluid-attenuated inversion recovery (FLAIR)). Infarct volume assessed by CTP will be calculated using NeuBrainCARE^®^ software (version 1.0, Neusoft, Beijing, China). All investigators responsible for enrollment will receive training on the imaging protocol and on the use of NeuBrainCARE^®^ software. For the baseline assessment, the infarction core volume will be considered to be those areas with a relative cerebral blood flow (rCBF) of less than 30%, as indicated by CTP. FLAIR images will be used to calculate the infarct volume at follow-up. If magnetic resonance imaging (MRI) at follow-up time is unavailable, computed tomography (CT) can be used.

The secondary efficacy outcomes include (1) early neurological improvement within 24 h: a decrease in NIHSS score of ≥8 points or NIHSS score of 0–2 within 24 h (2); neurological deterioration within 24 h: an increase in NIHSS score by ≥4 points within 24 h (3); NIHSS score at 24 h (4); proportion of successful reperfusion (mTICI 2b/3) (5); NIHSS score at 5 days (6); proportion of patients with functional independence (mRS 0–1) (7); mRS of 0–2 at 90 days (8); European quality of Life-5 dimensions (EQ-5D) at 90 days; and (9) proportion of patients with Barthel Index (BI) of 95–100 at 90 days. The safety endpoints will mainly include (1) intracerebral hemorrhage within 24 h (2), symptomatic intracerebral hemorrhage (ICH) within 24 h (3), new-onset atrial fibrillation within 5 days (4), pulmonary edema or congestive heart failure within 5 days (5), intracerebral hemorrhage within 5 days (6), decompressive craniectomy within 7 days (7), death within 90 days (8), adverse events (AEs), and (9) serious adverse events (SAEs).

### Sample size calculation

In historical controls, the mean growth in the post-EVT infarct core volume from baseline is 18.7 mL, with a variance of 9.7. Based on our pilot study (unpublished data), we assume that there will be a 30% reduction in infarct volume in the albumin group compared to the sham group. A sample size of 128 participants would provide the trial with 90% power at a two-sided alpha level of 0.05. Considering a 5% loss to follow-up, the total sample size estimate was 134 participants (67 per group).

### Statistical analysis

The main analysis will be performed in the intention-to-treat population. The primary efficacy outcome will be analyzed by a linear regression model or median regression model, depending on the distribution, to compare the change in infarct volume from baseline to day 5 between the intervention group and the control group, after adjusting for baseline ASPECTS, age, and baseline infarct core volume. The effect of the intervention is presented as a mean difference (or median difference) and 95% confidence intervals (CIs). For missing infarct volume data due to stroke-related death, the worst infarct growth will be imputed. Other missing data will be handled using multiple imputation.

For other outcomes, continuous variables will be analyzed using the same method as the primary efficacy outcome, and a mean difference (or median difference) with 95% CIs will be calculated. Binary variables will be estimated using a modified Poisson regression model, and the result will be presented as relative risk and corresponding 95% CIs. All analyses will be conducted at a two-sided level with a significance level of 0.05. Statistical tests will be performed using the R programming language (version 4.3.3).

### Visits and data collection

Study visit will be conducted at baseline, 24 h, day 5, day 7, or before discharge (the follow-up point will be determined by the earliest or latest event occurring between the two specified instances), and day 90 (Table 2). At baseline, computed tomography (CT), CT angiography (CTA), and CT perfusion (CTP) should be performed. On day 5 after randomization, MRI and MRA should be conducted. If MRI is unavailable, a CT scan may be performed. At baseline and during follow-up visits, clinical information will be collected using an electronic data capture system.

**Table 2:** Trial assessment flowchart.

	Baseline/Randomization	Immediately postoperative	24 h (±6 h)	Day 5 (±12 h)	Day 7 (±12 h) or at discharge	Day 90 (±7 days)
Inclusion/exclusion	×					
Informed consent	×					
Randomization	×					
Demographic data	×					
Medical history	×					
Vital signs	×	×	×	×	×	
Physical examination	×	×	×	×	×	
Previous medication	×					
NIHSS score	×		×	×	×	
Prestroke mRS	×					
Laboratory tests	×					
Brain CT plus CTP/MRI	×					
CTA/MRA	×			×		
MRI/CT				×		
DSA		×				
ASPECTS	×					
mTICI score		×				
mRS						×
Concomitant medication		×	×	×	×	×
Barthel index						×
EQ-5D						×
AE/SAE		×	×	×	×	×

CT, computed tomography; CTP, computed tomography perfusion; MRI, magnetic resonance imaging; CTA, computed tomography angiography; MRA, magnetic resonance angiography; DSA, digital subtraction angiography; ASPECTS, Alberta Stroke Program Early Computed Tomography Score; mTICI, modified Thrombolysis in Cerebral Infarction; mRS, Modified Rankin Scale; EQ-5D, EuroQol Five Dimensions Questionnaire; AE, adverse event; SAE, serious adverse event.

### Data management and quality control

The Data Safety Monitoring Board (DSMB) monitors the schedule and progress of the trial to ensure patient safety. The DSMB consists of a neurologist, a neuroradiologist, and a methodologist. These experts are not allowed to participate in the trial. The responsibilities of the DSMB include reviewing data completeness and safety, revising the trial protocol, and terminating the trial. The DSMB reviews all SAEs in a blinded manner, adjudicates their unexpectedness and relatedness to the study drug, and assesses adverse outcome events.

## Discussion

Our trial aimed to evaluate the safety and efficacy of albumin intravenous infusion for AIS patients who are receiving EVT. The study was designed with lessons learned from previous studies that failed to prove the effectiveness of the treatment (17). First, our trial ensures that all enrolled patients will undergo EVT and assess recanalization rates. Second, in the ALIAS trial, patients treated with albumin experienced a higher incidence of pulmonary edema and congestive heart failure compared to those treated with saline. According to the results of our pilot study, administering an albumin dose of 2 g/kg (as in the ALIAS trial) as four separate injections of 0.5 g/kg, given continuously over 4 days, results in a greater reduction in infarct volume and fewer adverse reactions. We anticipate that this modified dosing strategy will reduce the likelihood of pulmonary edema and congestive heart failure.

The ARISE trial will enroll patients with an infarct core volume of less than 100 mL, encompassing both small and large core infarcts. The inclusion of patients with a small infarct core volume is consistent with numerous studies, such as the ACTION II trial (Natalizumab in acute ischemic stroke) and the ApTOLL study, in patients with AIS (18, 19). Patients with large core infarcts are more prone to developing cerebral edema, which can lead to further ischemic damage (20). Albumin increases plasma colloid osmolality and has been widely supported in animal studies to reduce cerebral edema and improve neurological prognosis (14). Including patients with large core infarct volumes will provide an answer to the question of whether albumin is effective for patients with large core infarct volumes (20). The benefits of expanding the time window for patients to use neuroprotective medications are worth waiting for. Therefore, the ARISE study sets the treatment window at 24 h. Subsequent subgroup analyses may focus on age, infarct volume, last known good status to randomization time, NIHSS score, and stroke type.

The primary endpoint of the study is the change in infarct volume from baseline to day 5 in the intervention versus control groups. An increase in infarct volume (instead of clinical assessment at 3 months) was chosen because this biomarker is recommended by the Academic Industry Roundtable on Stroke Care and the Stroke Imaging Study (21, 22). It is an objective outcome and may be closely associated with clinical outcomes.

## Conclusion

To sum up, if the study outcomes are positive, they will provide significant evidence to support a phase 3 trial assessing the efficacy of albumin for AIS patients.
